# Visceral fat: the hidden culprit behind thoracolumbar surgery infections

**DOI:** 10.3389/fsurg.2025.1606944

**Published:** 2025-07-15

**Authors:** Dan Su, Ruiling Wang, Jucai Li, Xiaohui An, Lingling Sun, Yi Cui, Di Zhang

**Affiliations:** Department of Spinal Surgery, Third Hospital of Hebei Medical University, Shijiazhuang, China

**Keywords:** vsceral fat area (VFA), surgical site infection (SSI), thoracolumbar surgery, obesity, infection risk factors

## Abstract

**Objective:**

This study aimed to explore the relationship between visceral fat area (VFA) and the risk of surgical site infection (SSI) after thoracolumbar posterior surgery.

**Methods:**

A retrospective analysis was conducted on 1,491 patients who had undergone posterior thoracolumbar surgery from January 1, 2022, through May 30, 2023. Inclusion criteria were age ≥18 years, undergoing thoracolumbar posterior surgery, and having complete clinical data with a follow-up duration exceeding 1 year. Exclusion criteria included minimally invasive surgery, preoperative infections, traumatic skin injuries, combined tumors, and patients with long-term steroid use or immune system diseases. VFA was measured using CT scans, and patients were categorized based on VFA ≥100 cm^2^ as having visceral fat obesity. The incidence of SSI was assessed according to the CDC criteria. Logistic regression analysis was used to identify risk factors for SSI.

**Results:**

The incidence of SSI was 2.4% (36 out of 1,491 patients). Multivariate logistic regression analysis showed that VFA was the most significant predictor of SSI [*P* < 0.001; Exp(B) = 1.026; 95% CI, 1.013–1.040], indicating a 2.6% increased infection risk per 1 cm^2^ increase in VFA. Other significant risk factors included BMI [*P* = 0.024; Exp(B) = 1.138; 95% CI, 1.018–1.273]. Patients with visceral fat obesity had a significantly higher infection rate (5.7% vs. 1.2%, *P* < 0.001).

**Conclusion:**

VFA is a significant risk factor for SSI following thoracolumbar posterior surgery. Preoperative assessment of VFA can help identify high-risk patients and guide preventive measures to reduce SSI incidence and improve surgical outcomes.

## 1 Introduction

Surgical site infection (SSI) is one of the early complications of orthopedic surgery. It is common in thoracolumbar posterior surgery, and its incidence ranges from 0.72% to 8.7% (1–3). SSI can be divided into early infection and delayed infection according to the time of occurrence. SSI can cause refractory low back pain, affect early functional exercise, and reduce postoperative life quality, having an incidence of 0.2%–6.7% (4, 5). In particular, when combined with severe deep infection, repeated degenerative surgery may be required or even lead to fixation failure (6), thus greatly increasing the length of hospital stay and costs of patients. The late infection has a low incidence; however, most patients need a second operation to remove the internal fixation (7, 8). Therefore, the prevention of surgical site infection in the thoracolumbar posterior approach is of great significance.

Current evidence indicates that risk factors for postoperative infection following spinal surgery encompass diabetes, hypoproteinemia, prolonged surgical duration, and increased subcutaneous fat thickness (8–10). Notably, obesity and diabetes are well-established high-risk factors (11). However, the assessment of obesity in patients varies across studies, with methods including body mass index (BMI), waist circumference, subcutaneous fat thickness, and abdominal visceral fat area. Abdominal obesity, defined as a visceral fat area(VFA) ≥ 100 cm^2^, is a cardinal feature of metabolic syndrome (12, 13). It may influence postoperative wound healing and infection risk through mechanisms such as the release of pro-inflammatory cytokines, induction of insulin resistance, and hyperglycemia (14). Moreover, it provides a more accurate reflection of the patient's lipid metabolism disorder (15, 16).

However, due to the workload and technical challenges associated with measuring visceral fat, previous research on visceral fat has been limited, and the relationship between abdominal visceral fat and infection remains unclear. With the advancement of artificial intelligence and imaging software, the method of measuring abdominal fat using CT has become well-established (17–19). CT-based quantification of VFA can serve as the “gold standard” for diagnosing abdominal obesity, revealing the correlation between visceral fat accumulation and postoperative infection after thoracolumbar surgery, and providing a new target for preoperative intervention.

This study investigated the correlation between VFA and postoperative infection after thoracolumbar surgery, evaluating the significance of VFA in predicting postoperative infections.

## 2 Methods

A total of 1,491 patients who underwent thoracolumbar posterior surgery in the Department of Spinal Surgery at the Third Hospital of Hebei Medical University between January 1, 2022, to May 30, 2023. were included in this study. The inclusion criteria were as follows: (1) age ≥18 years old; (2) patients undergoing thoracolumbar posterior surgery; (3) complete clinical data and follow-up duration exceeding 1 year. The exclusion criteria were: (1) minimally invasive surgery: surgeries completed through various approaches and endoscopy. All patients included in this study were deemed unsuitable for minimally invasive surgery after evaluation by three senior spine surgeons, as well as those who were unwilling to undergo minimally invasive surgery or who explicitly requested thoracolumbar posterior spinal surgery. (2) preoperative spinal or other infections; (3) preoperative traumatic skin injuries involving the surgical area; (4) combined tumors; (5) patients with long-term steroid use or rheumatologic and immune system diseases, and those with malignant tumors or requiring surgical treatment for tumors in the vertebral body or vertebral canal. If internal fixation was required during the operation, prophylactic antibiotics should be administered preoperatively, and antibiotics should continue to be used to prevent infection within 24 h after surgery. We typically administer 1 g of cefazolin sodium prophylactically once before surgery and every 6 h within 24 h after surgery to control infection.

### 2.1 Observation index

**Figure 1:**
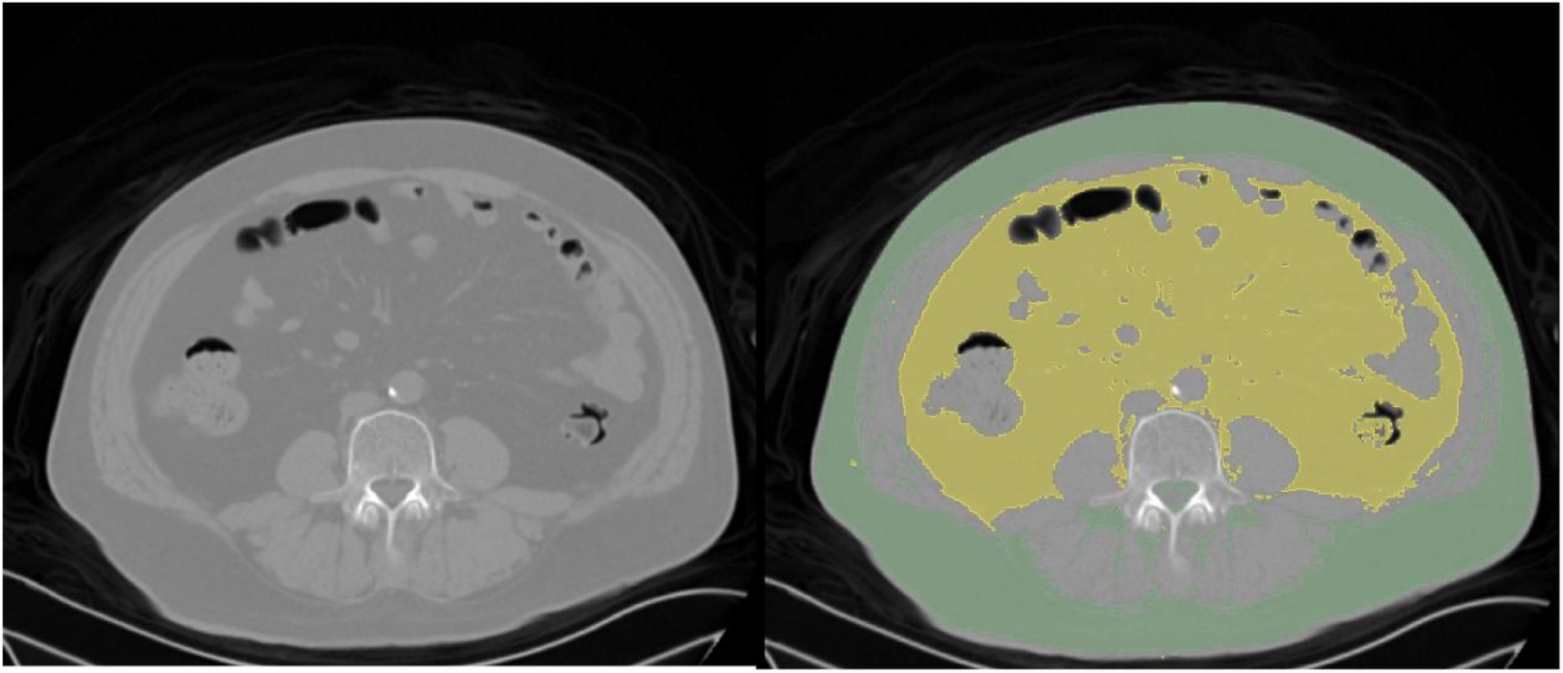
CT scan images from a 60-year-old male patient with a L5 vertebral slip. Left panel: Original CT image displaying the abdominal cross-section. Right panel: Processed CT image with subcutaneous fat highlighted in green and visceral fat in yellow.

The basic clinical information of the patients was recorded, including age and sex, BMI, smoking, alcohol use, underlying diseases, and surgical history. The surgical method, operative time, amount of blood loss, incision length, duration of drain placement, presence or absence of blood transfusion, and presence or absence of dural tear were also documented. The distance from the epidermis to the lamina was measured based on the radiographic results of the patients. All patients underwent lumbar CT scans within 1 week before surgery. Using the 3D Slicer software, the abdominal fat area was measured at the level of the umbilicus, with the Hounsfield unit (HU) value set between −190 and −30 HU (20, 21). Visceral fat type was defined as a VFA greater than 100 cm^2^ (Figure 1).

According to the Centers for Disease Control and Prevention (CDC), SSI was confirmed if one or more of the following 4 aspects were met (22): (1) the surgical wound showed redness, swelling, heat, pain, fluctuation, or abscess and purulent; (2) bacterial cultivate of wound secretion was positive; (3) in patients undergoing re-debridement, intraoperative lavage fluid or tissue bacterial cultivate was positive; (4) confirmed laboratory blood routine, C-reactive protein, procalcitonin, MRI or histopathological examination.

### 2.2 Statistical analysis

SPSS 22.0 (IBM, Armonk, NY, USA) statistical software was used for analysis, and the test level was *α* = 0.05. The measurement data were compared between the two groups and analyzed by Independent sample *t*-test or Mann–Whitney *U*-test according to their conformity with normal distribution and homogeneity of variance. The counting data were analyzed by the Chi-square test. The factors with *p* < 0.05 in univariate logistic regression were included in the multivariate logistic regression model. Multivariate Logistic regression was used to analyze risk factors for thoracolumbar posterior surgical infection and to calculate odds ratio (OR) and 95% confidence interval (95%CI).

## 3 Results

### 3.1 Baseline characteristics

This study included 1,491 patients, of whom 857 were male (57.5%) and 634 were female (42.5%), with a mean age of 52.4 years (range, 18–85 years). The mean BMI was 26.7 (range, 15.91–38.55), and 24.8% of patients were obese (BMI ≥ 28). Comorbidities included diabetes in 19.8%, hypertension in 32.1%, smoking history in 23.4%, and alcohol use in 31.2%. Surgical characteristics included a mean operative time of 72.56 min (range, 50–176 min), mean incision length of 6.85 cm (range, 4–18 cm), mean blood loss of 310.73 ml (range, 50–1,200 ml), and mean postoperative drainage volume of 339.81 ml (range, 50–800 ml) (Table 1).

**Table 1:** Characteristics between the infected and Non-infected groups.

Characteristic	SSI (*n* = 36)	No- SSI (*n* = 1,455)	*P*-value
Age	50.06 ± 8.695	52.48 ± 13.98	0.301
Sex (male/female)	24/12	833/622	0.259
BMI (kg/m^2^)	30.08 ± 6.43	26.62 ± 3.42	< 0.001
Diabetes (Yes/No)	11/25	284/1,171	0.101
Hypertension (yes/no)	13/23	466/989	0.604
Smoking (yes/no)	7/29	342/1,113	0.57
Alcohol Use (yes/no)	11/25	454/1,001	0.934
Surgical Site (Lumbar/Thoracic/Thoracolumbar)	26/8/2	1,092/216/147	0.361
Surgical Type (internal fixation/non-internal fixation)	35/1	1,307/148	0.144
Operative time (minutes)	73.94 ± 19.86	72.53 ± 18.97	0.659
Incision length (cm)	7.31 ± 2.47	6.84 ± 2.06	0.185
Blood loss (ml)	268.06 ± 175.72	311.79 ± 221.77	0.241
Postoperative drainage volume (ml)	336.111 ± 247.17	339.897 ± 253.85	0.93
SFT(cm)	2.51 ± 1.27	2.10 ± 0.88	0.005
VFA (cm^2^)	111.46 ± 54.37	72.83 ± 33.88	< 0.001

BMI, body mass index; SFT, subcutaneous fat thickness; VFA, visceral fat area.

### 3.2 Comparison of characteristics between viscerally obese and non-obese patients

Characteristics of patients with visceral obesity (VFA >100 cm^2^) and non-obese patients were compared (Table 2). Viscerally obese patients had significantly higher BMI, subcutaneous fat thickness, and VFA (all *P* < 0.001). The infection rate was also significantly higher in viscerally obese patients (5.7% vs. 1.2%, *P* < 0.001), suggesting that visceral obesity is an important risk factor for postoperative infection (Table 2).

**Table 2:** Characteristics between patients with visceral Fat obesity and Non-obese patients.

Characteristic	Visceral fat obesity (*n* = 405)	Non-visceral fat obesity (*n* = 1,086)	*P*-value
Age	52.16 ± 12.98	52.52 ± 14.21	0.658
Sex (male/female)	246/159	611/475	0.120
BMI (kg/m^2^)	28.67 ± 3.44	25.98 ± 3.31	**<0**.**001**
Diabetes (yes/no)	101/304	194/892	**0.002**
Hypertension (yes/no)	144/261	335/751	0.083
Smoking (yes/no)	104/301	245/841	0.206
Alcohol Use (yes/no)	137/268	328/758	0.179
Surgical Site (Lumbar/Thoracic/Thoracolumbar)	297/63/45	821/161/104	0.606
Surgical type (internal fixation/non-internal fixation)	365/40	977/109	0.927
Operative time (minutes)	71.83 ± 17.54	72.84 ± 3.31	0.360
Incision length (cm)	6.77 ± 2.00	6.89 ± 2.10	0.328
Blood loss (ml)	296.296 ± 219.72	316.114 ± 221.09	0.123
Postoperative drainage volume (ml)	333.95 ± 264.92	341.99 ± 256.14	0.586
SFT (cm)	2.37 ± 0.93	2.01 ± 0.86	**<0**.**001**
VFA (cm^2^)	119.41 ± 13.90	56.74 ± 23.27	**<0**.**001**
Infection(yes/no)	23/382	13/1,073	**<0**.**001**

BMI, body mass index; SFT, subcutaneous fat thickness; VFA, visceral fat area.

Bold values indicate statistical significance (*P* < 0.05).

### 3.3 Incidence of infection

Among the 1,491 patients, 36 cases of infection occurred, resulting in an infection rate of 2.4%. The distribution of baseline characteristics such as age, sex, BMI, surgical site, and surgical type between the infected and non-infected groups is shown in Table 1.

### 3.4 Univariate and multivariate analysis

Univariate analysis identified BMI (*P* < 0.001), subcutaneous fat thickness (*P* = 0.005), and VFA (*P* < 0.001) as potential risk factors for infection. Multivariate logistic regression analysis showed that VFA was the most significant predictor of infection [*P* < 0.001; Exp(B) = 1.026; 95% CI, 1.013–1.040], indicating a 2.6% increased infection risk per 1 cm^2^ increase in VFA. BMI was also significantly associated with infection [*P* = 0.024; Exp(B) = 1.138; 95% CI, 1.018–1.273]. Other factors, including age, sex, diabetes, hypertension, smoking, and alcohol use, were not statistically significant. Backward stepwise analysis further revealed that surgical type was a risk factor in the model (*P* = 0.171), while subcutaneous fat thickness was excluded as it did not contribute to model accuracy. See Table 3 for details.

**Table 3:** Multivariate logistic regression With outcome being SSI.

Variable	Coefficient (B)	Exp(B)	95% CI for Exp(B)		*p*	Coefficient (B)	Exp(B)	95% CI for Exp(B)		*p*
			Lower	Upper				Lower	Upper	
Age	−.016	.984	.947	1.023	.420					
Sex (female/male)	.038	1.039	.362	2.976	.944					
BMI	.129	1.138	1.018	1.273	.024	.124	1.132	1.027	1.248	.013
Diabetes	.029	1.029	.464	2.283	.944					
Hypertension	.069	1.072	.520	2.210	.851					
Smoking	−.310	.733	.306	1.756	.486					
Alcohol use	−.154	.857	.402	1.828	.690					
COPD	−17.838	.000	.000	.	.998					
Coronary heart disease	.204	1.227	.406	3.706	.717					
History of stroke	−.376	.687	.157	3.002	.618					
Surgical Site (Lumbar/Thoracic/Thoracolumbar)	−.122	.886	.513	1.529	.663	−1.404	.246	.033	1.832	.171
Surgical type (internal fixation/non-internal fixation)	−1.489	.226	.030	1.711	.150					
Incision length (cm)	.071	1.074	.920	1.253	.367					
Operative time	.010	1.010	.990	1.031	.335					
Blood loss	−.001	.999	.997	1.001	.302					
Postoperative drainage volume	.000	1.000	.999	1.002	.913					
SFT	−.042	.959	.629	1.460	.844					
VFA	.026	1.026	1.013	1.040	.000	.026	1.027	1.014	1.040	<0.001

BMI, body mass index; SFT, subcutaneous fat thickness; VFA, visceral fat area; COPD, chronic obstructive pulmonary disorder.

### 3.5 Correlation between visceral fat and infection

**Figure 2:**
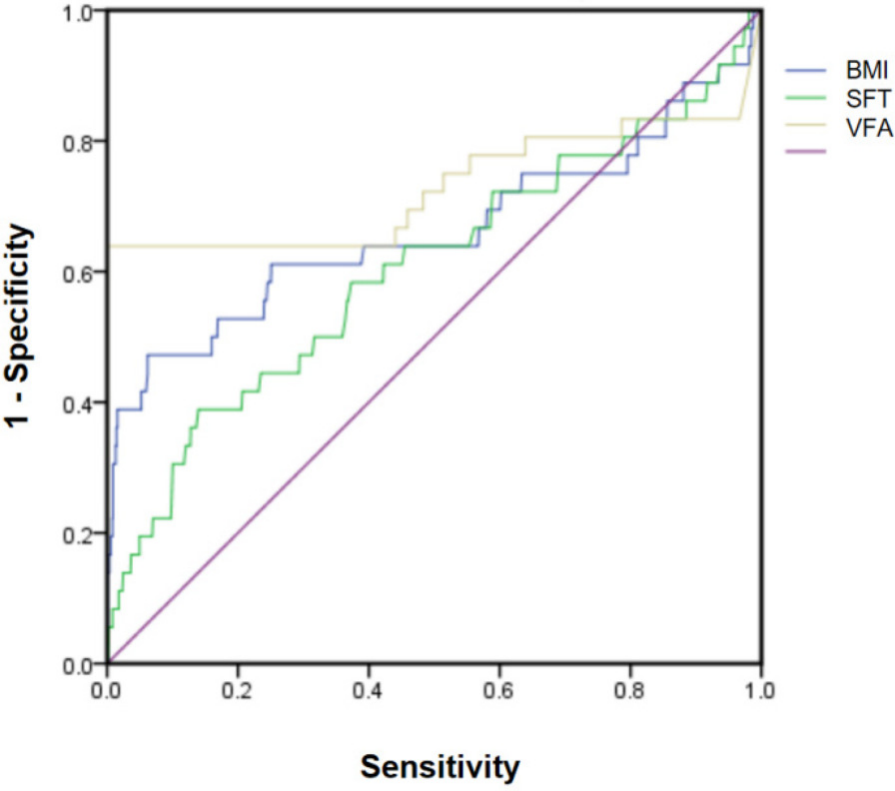
Receiver operating characteristic (ROC) curves comparing the diagnostic performance of body mass index (BMI), subcutaneous fat thickness(SFT), and visual fat assessment (VFA).

Table 4 presents the results of the correlation analysis between BMI, subcutaneous fat thickness, VFA, and infection. BMI was positively correlated with both subcutaneous fat thickness and VFA. The correlation between VFA and infection was strong (*P* < 0.01). The ROC curve analysis also demonstrated that VFA had the largest area under the curve (Figure 2).

**Table 4:** Correlation analysis of obesity indicators with infection.

Variable	BMI	Subcutaneous fat thickness	VFA	Infection
BMI	1	0.434**	0.451**	0.150**
Subcutaneous Fat Thickness	0.434**	1	0.233**	0.072**
VFA	0.421**	0.233**	1	0.170**
Infection	0.150**	0.072**	0.170**	1

** indicates *P* < 0.01.

## 4 Discussion

SSI is one of the common complications of open thoracolumbar posterior surgery. Given that thoracolumbar surgery often involves important neural structures such as the spinal cord and cauda equina, postoperative infections can lead to more complex conditions. Internal fixation is a commonly used and effective method in spinal surgery to enhance immediate stability. However, if SSI occurs, it may lead to failure of internal fixation and even necessitate the removal of the fixation devices. Early removal of internal fixation devices may increase the risk of pseudoarthrosis formation (23). SSI can also lead to prolonged hospital stays, increased hospital costs, delayed postoperative functional recovery, persistent low back pain, and decreased quality of life after surgery. Therefore, preventing SSI is an important measure to improve the therapeutic efficacy of thoracolumbar surgery (5–7). This study explored the risk factors for postoperative infection after thoracolumbar surgery through univariate analysis and logistic regression analysis. For the first time, VFA was introduced as a risk factor for postoperative infection in thoracolumbar surgery. This provides a theoretical basis for the prevention of SSI and offers references for early preventive measures, reducing the incidence of infection, and improving clinical outcomes.

### 4.1 Diabetes and infection

Diabetes has been confirmed as an independent risk factor for SSI following lumbar posterior surgery. Chronic hyperglycemia may lead to resting neutrophil dysfunction, thereby reducing the basal inflammatory response (24). Moreover, diabetes can also lead to systemic microangiopathy and local tissue ischemia, which in turn suppress the immune system, including the phagocytic function of granulocytes and the activity of lymphocytes, thereby increasing the incidence of SSI (25–27). Golinvaux et al. (28) observed 15,480 patients with non-insulin-dependent diabetes and 787 patients with insulin-dependent diabetes. They found that the risk of SSI in patients with insulin-dependent diabetes was 1.9 times higher than that in patients without diabetes. Zach et al. (3) also pointed out that high blood glucose levels and poor glycemic control (high variability and postoperative mean values) may be etiological factors for SSI. Although the results of this study did not show a statistically significant difference between diabetes and postoperative infection, this may be related to better control of diabetes or a smaller sample size of infections.

### 4.2 BMI and infection

A high BMI has been confirmed to be positively correlated with the incidence of early SSIs (29, 30). In patients with a high BMI, the subcutaneous fat layer is thicker, which makes it easier for cavities to form due to liquefaction and necrosis after surgery, providing more favorable conditions for bacterial proliferation (31). Additionally, the thick fat layer may also lead to dilution of antibiotic concentrations, reducing local drug levels and weakening the effectiveness of anti-infection measures, thereby further increasing the risk of postoperative wound infection (32). This study also reached a similar conclusion: for such patients, it is important to actively screen for underlying diseases, enhance postoperative care, and implement more comprehensive infection prevention measures (33, 34).

### 4.3 Fat distribution and infection

In this study, the relationship between subcutaneous fat thickness and postoperative infection did not reach statistical significance (*P* = 0.05), but it approached the level of significance, suggesting that it may be a potential risk factor for postoperative infection. Previous studies have shown that an increased subcutaneous fat thickness is closely related to the risk of postoperative infection. For example, Mehta et al. (35) found that subcutaneous fat thickness and the distance from the lamina to the skin are more accurate predictors of postoperative infection than BMI. Moreover, excessive subcutaneous fat may lead to difficulties in surgical field exposure, prolonged operative time, local tissue ischemia and necrosis, and the formation of dead space after surgery, thereby increasing the risk of infection (36). However, the lack of significant difference in subcutaneous fat thickness in this study may be related to the insufficient sample size or the specificity of the surgical site. Future studies may further investigate the impact of subcutaneous fat thickness on postoperative infection and conduct a comprehensive evaluation in combination with other factors.

### 4.4 Operative time and infection

Although there was no statistical difference between operative time and infection in this study, this may be related to the relatively short average operative time of the patients included in this study. Previous studies have described that operative time is closely related to SSI (37). Possible reasons include: (1) Prolonged operative time leads to increased exposure time of the incision, thereby increasing the probability of bacterial colonization of the incision; (2) Long-term muscle traction during surgery causes muscle damage and prolonged muscle ischemia, thereby expanding the range of necrotic muscle; (3) As the operative time increases, the concentration of prophylactically administered antibiotics gradually decreases; (4) The trauma and bleeding caused by surgery lead to a stress response. Moreover, prolonged exposure time makes it easier for bacteria to invade the body, and the rate of ectopic growth of intestinal flora increases, which more easily leads to SSI (31, 38). Therefore, in long-duration surgeries, more attention should be paid to aseptic operations, avoiding violent maneuvers, and moderate traction of muscle tissues. In addition, when the operative time exceeds 3 h, intraoperative antibiotics should be used to maintain drug concentrations in the blood.

### 4.5 Visceral Fat and infection

This study particularly focused on the relationship between visceral fat area and infection. The results showed that VFA was positively correlated with the incidence of infection (*P* < 0.001, *r* = 0.170). The incidence of infection in patients with visceral fat obesity (VFA > 100 cm^2^) was 5.7%, significantly higher than that in patients without visceral fat obesity (1.2%, *P* < 0.001). This indicates that visceral fat may play an important role in the occurrence of infection. Visceral fat may affect postoperative wound healing and infection risk through mechanisms such as the release of pro-inflammatory factors, induction of insulin resistance, and hyperglycemia. An increasing number of studies have focused on the impact of VFA on the body. For example, a study by Amy Z. B et al. (39) indicated that visceral fat is a risk factor for periprosthetic joint infection after total hip and knee arthroplasty. Another study by Peng Zhang et al. (40) revealed that patients with visceral fat obesity are more likely to suffer from osteoporotic vertebral compression fractures. Moreover, the measurement of VFA has become well-established. CT-based quantification of VFA can serve as the “gold standard” for diagnosing abdominal obesity and provides a new target for preoperative intervention.

Currently, the molecular mechanisms by which VFA affects human health and influences the prognosis of postoperative patients are not yet clear. However, clinical studies have already shown significant variability. Future research could further explore the mechanisms through which visceral fat influences the risk of postoperative infection. Additionally, a comprehensive risk assessment model could be established, integrating multiple infection-predictive factors to more accurately predict the risk of postoperative infection and provide clinicians with a preoperative risk assessment tool (41).

### 4.6 Limitation

This study has several limitations. First, it is a single-center retrospective study, with all patients recruited from the same hospital. This may limit the generalizability of the results due to potential biases related to geographic location and healthcare settings. Second, although the sample size is relatively large, the incidence of postoperative infection is low (2.4%), which may affect the statistical power and necessitate cautious interpretation of the significance of some factors. Additionally, the study did not differentiate between types of diabetes or treatment modalities and did not explore the underlying mechanisms linking visceral fat to infection. Future research could employ a multicenter design, increase the sample size, and incorporate prospective studies to further validate the findings.

## 5 Conclusion

This study, through the analysis of clinical data from patients undergoing thoracolumbar posterior surgery, has revealed several risk factors associated with postoperative infection, including high BMI, VFA, diabetes, previous surgical history, and operative time. These findings provide important references for clinicians to assess the risk of infection preoperatively, develop preventive measures, and optimize surgical strategies. Future research should further explore the interaction mechanisms among these factors and validate the effectiveness of preventive measures based on these risk factors, in order to reduce the incidence of postoperative infection after thoracolumbar surgery and improve patient outcomes.

## Data Availability

The raw data supporting the conclusions of this article will be made available by the authors, without undue reservation.
